# Physical activity as an aid to smoking cessation during pregnancy (LEAP) trial: study protocol for a randomized controlled trial

**DOI:** 10.1186/1745-6215-13-186

**Published:** 2012-10-04

**Authors:** Michael Ussher, Paul Aveyard, Isaac Manyonda, Sarah Lewis, Robert West, Beth Lewis, Bess Marcus, Adrian H Taylor, Pelham Barton, Tim Coleman

**Affiliations:** 1 Division of Population Health Sciences and Education, St George’s University of London, Cranmer Terrace, London, SW17 ORE, UK; 2 Department of Primary Care Health Sciences, University of Oxford, Radcliffe Observatory Quarter, Woodstock Road, Oxford, OX2 6GG, England; 3 Division of Epidemiology and Public Health and UK Centre for Tobacco Control Studies, University of Nottingham, Nottingham, NG5 1 PB, UK; 4 Health Behaviour Research Centre, Department of Epidemiology and Public Health, UCL, Gower Street, London, WC1E 6BT, UK; 5 School of Kinesiology, University of Minnesota, Minneapolis, MN 55455, USA; 6 Department of Family and Preventive Medicine, University of California, San Diego, CA, 92093-0628, USA; 7 School of Sport and Health Sciences, Sport and Health Sciences, University of Exeter, Exeter, EX1 2 LU, UK; 8 Health Economics, School of Health and Population Sciences, University of Birmingham, Birmingham, B15 2TT, UK; 9 Division of Primary Care and UK Centre for Tobacco Control Studies, University of Nottingham, Nottingham, NG5 1 PB, UK

**Keywords:** Smoking cessation, Pregnancy, Physical activity, Intervention, Randomized controlled trial

## Abstract

**Background:**

Many women try to stop smoking in pregnancy but fail. One difficulty is that there is insufficient evidence that medications for smoking cessation are effective and safe in pregnancy and thus many women prefer to avoid these. Physical activity (PA) interventions may assist cessation; however, trials examining these interventions have been too small to detect or exclude plausible beneficial effects. The London Exercise And Pregnant smokers (LEAP) trial is investigating whether a PA intervention is effective and cost-effective when used for smoking cessation by pregnant women, and will be the largest study of its kind to date.

**Methods/design:**

The LEAP study is a pragmatic, multi-center, two-arm, randomized, controlled trial that will target pregnant women who smoke at least one cigarette a day (and at least five cigarettes a day before pregnancy), and are between 10 and 24 weeks pregnant. Eligible patients are individually randomized to either usual care (that is, behavioral support for smoking cessation) or usual care plus a intervention (entailing supervised exercise on a treadmill plus PA consultations). The primary outcome of the trial is self-reported and biochemically validated continuous abstinence from smoking between a specified quit date and the end of pregnancy. The secondary outcomes, measured at 1 and 4 weeks after the quit date, and at the end of pregnancy and 6 months after childbirth, are PA levels, depression, self-confidence, and cigarette withdrawal symptoms. Smoking status will also be self-reported at 6 months after childbirth. In addition, perinatal measures will be collected, including antenatal complications, duration of labor, mode of delivery, and birth and placental weight. Outcomes will be analyzed on an intention-to-treat basis, and logistic regression models used to compare treatment effects on the primary outcome.

**Discussion:**

This trial will assess whether a PA intervention is effective when used for smoking cessation during pregnancy.

**Trial registration:**

ISRCTN48600346

## Background

Maternal smoking during pregnancy is the major preventable cause of poor health outcomes for women and their babies. Smoking during pregnancy causes substantial harm to mothers and infants, increasing the risks of post-natal depression, miscarriage, stillbirth, prematurity, low birth weight, perinatal mortality and morbidity, asthma, attention deficit disorder, learning difficulties, and obesity [1-6]. Smoking also presents immediate risks for the mother, including placental abruption [7], as well as the longer-term risks reported for smokers in general. Smoking in pregnancy is a major public health problem in high-income countries; in the USA, 14% of pregnant women smoke throughout their pregnancy [8]; in the UK, 12% of pregnant women smoke [9], although a figure of 40% has been reported in deprived areas [10]. Only around 25% of pregnant smokers stop for even part of their pregnancy, and of these, around two thirds re-start smoking post-natally [11]. The most effective smoking-cessation therapy in non-pregnant smokers is a combination of behavioral support plus nicotine replacement therapy (NRT), bupropion, or varenicline [12-14]. However, the safety and efficacy of NRT during pregnancy is not yet known [15], thus many pregnant women are reluctant to use NRT [16], and other smoking-cessation medications are contraindicated during pregnancy [17]. Behavioral support can increase smoking cessation rates in pregnancy by around 6% [18], and there is a need to identify other non-pharmacological interventions that are effective for smoking cessation during pregnancy.

Pharmaceutical aids for quitting are thought to work mainly through reducing cigarette cravings [14], and there is good evidence that PA moderates these cravings, therefore PA interventions have the potential to aid smoking cessation. A recent systematic review considered the evidence for PA aiding cessation for smokers in general [19]. Most of the 15 randomized controlled trials (RCTs) reviewed were insufficiently powered to detect a meaningful difference between the treatment groups, with seven of the trials having fewer than 25 participants in each treatment arm. Six adequately powered trials compared a group receiving a PA intervention as an adjunct to behavioral support with a group receiving behavioral support alone. Three of these studies showed significantly higher smoking abstinence rates in a physically active group versus a control group at end of treatment [20-22]. One of these studies also showed a benefit on abstinence for exercise versus control at the 3-month follow-up, and a benefit for exercise of borderline significance (relative risk (RR) = 2.19, *p* = 0.05) at the 12-month follow-up [20]. A further study showed significantly higher abstinence rates for the exercise group compared with control group at the 3-month follow-up, but not at the end of treatment or at the 12-month follow-up [23]. The latter study also found that those with higher levels of exercise adherence were significantly more likely to achieve abstinence at the end of treatment. The study with the most intensive PA intervention (sessions of supervised vigorous intensity exercise three times per week) showed the strongest effect on abstinence rates [20]. The other studies entailed PA interventions that were insufficiently intensive to ensure the desired levels of PA. It was concluded that PA levels need to be raised to at least 110 minutes a week to aid long-term abstinence, and supervised exercise sessions on two or more days per week are probably necessary to achieve this aim.

Physical activity could also aid smoking cessation during pregnancy. Moderate-intensity PA is recommended during pregnancy [24,25], and may represent an attractive aid to cessation for pregnant smokers who are reluctant to use NRT [16] or who wish to use it as an adjunct to NRT, and who fear post-cessation weight gain or who are concerned about weight and muscle-tone issues during post-partum [26]. Pregnant smokers have been shown to express interest in using PA to help them quit [27], and we have conducted two pilot studies to assess the feasibility of using PA for smoking cessation during pregnancy [28]. In the first study, 10 women recruited from antenatal clinics attended eight once-weekly sessions combining individual behavioral support for smoking cessation with 30 minutes of brisk walking and PA consultations. All the women requested a more extensive PA program; despite this, five women achieved continuous abstinence (validated by carbon monoxide (CO) levels) to the end of pregnancy. In the second pilot, a more intensive intervention entailed 22 women receiving 9 weeks of individual smoking cessation support, plus 15 sessions of supervised exercise (brisk treadmill walk or stationary cycling) and PA consultations. Three women maintained continuous (CO validated) abstinence to the end of pregnancy, attending on average 11 of the 14 exercise sessions. All the abstinent women achieved the target of 110 minutes of PA each week. Combining both pilots, 25% of the women (8/32) sustained continuous abstinence to the end of pregnancy. Our pilot work suggests that it is feasible to provide a PA intervention, combining supervised exercise and PA consultations, as an aid to smoking cessation during pregnancy.

### Aim

The aim of this paper is to describe the protocol for an RCT designed to evaluate an intervention for increasing PA as an aid for smoking cessation during pregnancy.

## Methods

### Study design

**Figure 1:**
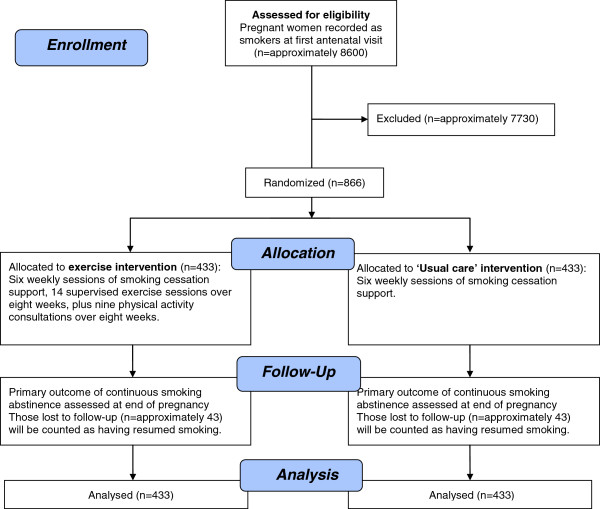
Consolidated Standards of Reporting Trials (CONSORT) flow diagram of the London Exercise And Pregnant smokers (LEAP) trial design.

The London Exercise And Pregnant smokers (LEAP) study is a pragmatic two-arm RCT into which pregnant women who smoke are recruited from antenatal clinics in National Health Service (NHS) trust hospitals (see Figure 1 for a flowchart of the LEAP study design). The study will compare the effectiveness on smoking cessation of individual behavioral support for smoking cessation plus a PA intervention, relative to individual behavioral support alone at the end of pregnancy.

### Ethics approval

Ethical approval for the study was given by Wandsworth Research Ethics Committee and research governance approval was obtained from each of the 13 hospital trusts recruiting to the study. Each participant is to provide written informed consent.

### Eligibility criteria

Exercise trials for smoking cessation often target those who report smoking 5 to 10 or more cigarettes a day. However, many pregnant smokers under-report the number of cigarettes they smoke [29], therefore, women are eligible if they report currently smoking at least one cigarette a day and report smoking at least five cigarettes a day before pregnancy. Participants are eligible if they are aged 16 to 50 years, 10–24 weeks pregnant (subject to confirmation that they have had a scan to show a viable pregnancy), prepared to quit smoking 1 week after enrolment, and confirm that they are able to walk continuously for at least 15 minutes. Women are excluded if they are unable to complete self-administered questionnaires in English (because of lack of resources for translators) or if they report having any medical condition that might be exacerbated by exercise. There are no documented contraindications to moderate-intensity exercise, but if the women have been advised by their doctor or midwife not to take exercise during pregnancy, if they have any complications during their pregnancy, or if they have any cautions for taking exercise [24,30], a consultant obstetrician and gynecologist at their hospital will be consulted to check that it is safe for them to participate. Participants joining the trial are monitored at each treatment session for cautions to exercise and for adverse events. Those with drug or alcohol dependence will also be excluded as the intervention described here is not sufficiently comprehensive to address the problems of women who have drug or alcohol dependence.

Although NRT is licensed for use in pregnancy, there is mixed evidence on its effectiveness in helping women quit smoking during pregnancy [15], and the most robust trial to date has found no evidence that it is effective [31]. In addition, many pregnant smokers prefer not to use NRT [16]. Allowing participants in the trial to use NRT might create confounding, and therefore, women who indicate that they wish to use NRT at the outset will be excluded. Our pilot work shows that pregnant smokers are willing to attempt to quit without using NRT as part of a research study [28]. Following the guidelines [17], those women who are unable to stop smoking after their quit day, and who express a clear wish to receive NRT, will be prescribed NRT by their general practitioner (GP). Use of NRT will be monitored throughout the intervention period with the following questions:

• Have you used any nicotine replacement therapy (NRT) this week?

• If yes, which type of NRT have you mainly used?

• How many days, approximately, have you used NRT in the past week?.

In addition, at the end of pregnancy follow-up participants will be asked the following questions:

• Besides the help we have given you, have you received any face-to-face support to stop smoking during your pregnancy?

• If yes, approximately how many sessions have you attended?.

The subjects’ GPs, midwives, and obstetricians will be informed of their patient’s participation in the trial.

### Sample size

A Cochrane review suggests that approximately 9% of women who are still smoking at the time of their first antenatal visit will stop smoking with usual care through to the end of their pregnancy, and a further 6% will stop as a result of a smoking cessation program using individual behavioral support [18]. Thus, in our control group we expect a smoking cessation rate of around 15% at the end of pregnancy. Combining our pilot studies, 25% (8/32) of participants in the treatment group sustained continuous smoking abstinence to the end of pregnancy. Therefore, in the trial we conservatively estimate an abstinence rate of 23% at the end of pregnancy in the treatment group, which would be similar to the effect shown for NRT with non-pregnant smokers [14]. We aim to recruit 433 women to each arm to detect the above absolute difference (8%) in smoking cessation rates between the groups at end of pregnancy with a two-sided significance level of 5% and power of 83%. This calculation is based on a χ^2^ test with Yates correction.

### Recruitment

Recruitment for the trial is taking place over a 44-month period, between April 2009 and November 2012, in 13 NHS hospital trusts in London and the south-east of England. During the trial, smoking status for all pregnant women will be recorded in the hospital computerized patient administration system at the first antenatal booking visit, which is typically at 9–14 weeks of gestation. At this time, the midwife will inform all women recorded as smokers that it is the hospital’s policy to telephone them to offer smoking-cessation support. This support would usually be offered by the primary care trust (PCT) stop-smoking advisor, but during the period of recruitment to the study, a trial researcher will telephone the women. Those who are interested in receiving help with quitting will be invited to join the trial or be referred to the PCT. Information for the first antenatal appointment will be accompanied by a flyer inviting women who smoke to join the study. Those women expressing interest in volunteering will be screened for eligibility by a telephone interview, and eligible women will be sent a patient information sheet. As an incentive for recruitment, and to increase attendance rates, all women attending at least 80% of their treatment sessions will be entered into an annual lottery with three annual prizes of £100 shopping vouchers.

### Randomization

At the first visit, eligible and consenting patients will be individually randomized to one of the two treatment arms based on a computer-generated pseudo-random code using random permuted blocks of randomly varying size, created by the Nottingham University Clinical Trials Unit (CTU) and held on a secure server. The randomization will be stratified by center, and block randomization will be used to ensure good balance between intervention and control within centers and thus minimize the risk of any ‘center effect’ influencing the outcome. Access to the sequence will be confined to the CTU data manager. Allocation to treatment arms will be in the ratio 1:1, and investigators will access the treatment allocation for each participant by means of a remote, secure internet-based randomization system developed and maintained by the CTU. Allocation will be concealed from the patient until they have completed all baseline assessments. The sequence of treatment allocations will be concealed until interventions have all been assigned and recruitment, data collection, and laboratory analyses are complete.

### Interventions

The interventions (Table 1, Table 2) follow CONSORT guidelines for non-pharmacologic interventions [32,33]. The same therapists, including research midwives, nurses, and psychologists, will deliver both the control and exercise interventions. Two therapists operate at three of the recruitment sites, and the remaining 10 sites have been assigned a single therapist. Delivery of the interventions will be standardized by training and by the therapists following the supplied manuals (see Additional file 1, see Additional file 2). The initial competence of the therapists will be assessed by the trial manager observing role-play scenarios during training. The fidelity of the interventions will be checked during the first 6 months by regular observations (at least five intervention sessions) by the trial manager. All sessions will be face-to-face and one-to-one, and will be delivered in a private room at the hospital or in a community health center. Social cognitive (learning) theory [34] is the theoretical basis for the interventions. This theory recognizes the interplay of individual factors (for example, self-efficacy to quit smoking or increase PA) and social/environmental factors (for example, social support) on health behavior change. For each session that they attend, the women are paid £7 for their travel expenses.

**Table 1:** Behavior change techniques (BCTs) used in the smoking-cessation consultations in this study

**Week**	**Session number**	**Session content**	**BCTs used (Michie categories**^**a**^**)**
1	Session 1 (1 week before quit day)	Explain the treatment, including timing of quit	RC4, BS4
		Measure expired CO and explain purpose	RC3
		Assess and discuss current and past smoking behavior	RI1
		Identify reasons for wanting and not wanting to quit	BM9
		Assess current motivation/confidence for quitting	R12
		Discuss past attempts at quitting	R13
		Prepare for the quit attempt	BM6, BS3
		Discuss use of social support	A2
		Advise on reducing smoking cues	BS8
		Advise subject to note the times when they are likely to lapse	BS6
		Facilitate relapse prevention planning and coping	BS2
		Identify barriers to quitting and address these barriers	BS1
		Emphasize choice (for example, when the participants take their final smoke)	RD2
		Provide information about the consequences of smoking during pregnancy	BM1, RC5
		Explain about quitting abruptly, rather than cutting down	BM10
		For all sessions:	
		Allow time for questions	RC2
		Summarize	RC9
		Use reflective listening	RC7
		Elicit participant’s views	RC8
		Build a general rapport	RC1
		Give praise for progress	BM7
		Tailor the interactions	RD1
2	Session 2 (quit day)	Look for reasons why the woman is a good prospect	BM2, BM3
		Explain about cigarette withdrawal symptoms and strategies for dealing with them	RC6
		Identify barriers to quitting and address these barriers	BS1
		Advise on avoiding social cues for smoking	BS11
		Advise on changing routine	BS7
		Advise on conserving mental resources	BS10
		Set graded tasks (for example, take 1 hour/day at a time)	BS9
3	Session 3 (1 week after quit day)	Check smoking status	BS5
		Assess withdrawal symptoms	R14
		Reassure about the norms for these symptoms	RC10, BM5
		Advise subject to monitor when they want to smoke	BS6
		Assess CO and give feedback about whether reading has reduced	BM11, BM3
		Discuss planning and coping strategies to prevent relapse	BS2
		If they, have relapsed ask them to commit to a new quit date	BM6
		Advise about use of NRT	A1
		Liaise with PCT about obtaining NRT	A3
		Encourage subject to see themselves as a non-smoker	BM8
		Remind them of lottery prize for attending all sessions	BM7
4	Session 4 (2 weeks after quit day) onwards	Assess CO	BM11
		Check smoking status	BS5
		If they are struggling offer further support from PCT	A5
		Discuss relapse prevention planning and coping strategies for after birth	BS2, BM8
		Emphasize importance of not having a single puff	BM6
		If subject has relapsed, set a new quit date, and review use of NRT	A4

CO, carbon monoxide; PCT, primary care trust; NRT, nicotine replacement therapy.

^**a**^**Michie categories are defined as follows**.

*Specific focus on the target behavior (B) and maximizing motivation (M).* BM1: provide information on consequences of smoking and smoking cessation. BM2: boost motivation and self-efficacy. BM3: Provide feedback on current behavior. BM5: provide normative information about others’ behavior and experiences: BM6: prompt commitment from the client there and then. BM7: Provide rewards contingent on effort or progress. BM8: strengthen ex-smoker identity. BM9: identify reasons for wanting and not wanting to stop smoking. BM10: explain the importance of abrupt cessation. BM11: measure CO levels.

*Maximizing self-regulatory capacity and skill (BS)*. BS1: facilitate barrier identification and problem-solving. BS2: facilitate relapse-prevention and coping. BS3: facilitate action-planning/develop treatment plan. BS4: facilitate goal-setting. BS5: prompt review of goals. BS6: prompt self-recording. BS7: advise on changing routine. BS8: advise on environmental restructuring. BS9: set graded tasks. BS10: advise on conserving mental resources. BS11: advise on avoiding social cues for smoking.

*Promoting adjuvant activities (A).* A1: advise on stop-smoking medication. A2: advise on/facilitate use of social support. A3: adopt appropriate local procedures to enable clients to obtain free medication. A4: ask about experiences of stop-smoking medication that the smoker is using. A5: give options for additional and later support.

*General aspects of interaction focusing on delivery of the intervention (RD)*. RD1: tailor interactions appropriately. RD2: emphasize choice.

*General aspects of interaction focusing on information gathering (RI).* RI1: assess current and past smoking behavior. RI2: assess current readiness and ability to quit. RI3: assess history of quit attempts. RI4: assess withdrawal symptoms.

*General aspects of interaction focusing on general communication (RC).* RC1: build general rapport. RC2: elicit and answer questions. RC3: explain the purpose of CO monitoring. RC4: explain expectations regarding treatment program: RC5: offer/direct toward appropriate written materials. RC6: provide information on withdrawal symptoms. RC7: use reflective listening. RC8: elicit client views. RC9: summarize information/confirm client decisions. RC10: provide reassurance.

**Table 2:** Behavior change techniques (BCTs) used in the physical activity (PA) consultations

**Week**	**Session number**	**Session content**	**BCTs used (Michie categories**^**a**^**)**
1	Session 1 (one week before quit day)	Review current PA and discuss PA benefits	1, 2
		Explain and demonstrate use of treadmill and pedometer	7, 21, 22, 26
		Check PA confidence levels using scaling questions	16
		All sessions:	
		Agree PA goals 10	
		Provide weekly PA and step-count diaries 16	
		Allow time for questions, summarize, use reflective listening, elicit participant’s views, build a general rapport	
		Give praise for effort and for achieving PA goals 12, 13	
1	Session 2	Review PA goals and effect of PA on cravings	7, 9, 10
		Complete cost-benefit analysis for increasing PA	2
		Identify PA barriers and problem solve	8
		Explain and demonstrate exercises in booklet	21, 22, 26
		Provide information on places to exercise	20
		Discuss time management and exercise habits	23, 38
		Plan social support	29
		Provide weekly PA diary and step-count diary	16
2	Session 3 (quit day)	Review PA goals, set heart-rate targets on treadmill	10
		Identify PA barriers and problem solve	8
		Provide weekly PA diary and step-count diary	16
		Check PA confidence levels with scaling questions	8
3	Session 4 (one week after quit day) onwards	Review PA goals, set heart-rate targets on treadmill	10
		Plan for relapse prevention/coping	35
		Review exercises in booklet	21, 22, 26
		Review social support	29
		Use imagery to encourage identity as an ‘exerciser’	34
		Provide weekly PA diary and step-count diary	16
		Reminder that sessions reduce to once a week for the last 2 weeks of the program	27
		Check PA confidence levels with scaling questions	8

^**a**^**Michie categories are defined as follows**.

1) Provide information on consequences of behavior in general. 2) Provide information on consequences of behavior to the individual. 7) Action-planning. 8) Barrier identification/problem-solving. 9) Set graded tasks. 10) Prompt review of behavioral goals. 12) Prompt rewards contingent on effort or progress towards behavior. 13) Provide rewards contingent on successful behavior. 16) Prompt self-monitoring of behavior. 20) Provide information on where and when to perform the behavior. 21) Provide instruction on how to perform the behavior. 22) Model/demonstrate the behavior. 23) Teach subject to use prompts/cues. 26) Prompt practice. 27) Use of follow-up prompts. 29) Plan social support/social change. 34) Prompt use of imagery. 35) Relapse-prevention/coping planning. 38) Time management.

#### Control group

Those in the control group will receive behavioral support for smoking cessation, which is generally provided via the NHS Stop Smoking Services to pregnant women as part of ‘usual care’. By extracting the elements of the intervention from written manuals and materials provided by the program (see Additional file 1), the contents of the intervention have been classified in accordance with the taxonomy of behavior-change techniques (BCTs) described by Michie and colleagues, and used in individual behavioral support for smoking cessation [35] (Table 1). The researchers delivering the intervention will be trained to NHS Centre for Smoking Cessation and Training standards during a 2-day course [36]. Participants will receive six once-weekly sessions of behavioral support, each session lasting for approximately 20 minutes. This begins 1 week before the quit date and ends 4 weeks after that date. A pregnancy-specific smoking cessation program is used [17], the contents of which are described in Table 1. The intervention incorporates all 43 BCTs for smoking cessation defined by Michie and colleagues [35], except for the BCT ‘provide rewards contingent on successfully stopping smoking’, although rewards will be offered to increase compliance (see Table 3). Continuing support will be offered to women who fail to quit or relapse to smoking.

**Table 3:** Financial incentives offered to trial participants

**Incentive occasion**	**Maximum financial incentive, GBP**	
	**Exercise group**	**Control group**
Annual lottery with three prizes of £100^**a**^ for attending at least 80% of treatment sessions	100	100
£7^**b**^ travel expenses for each session attended	98 (14 sessions)	42 (6 sessions)
£10^**a**^ for follow-up at end of pregnancy	10	10
£10^**a**^ for follow-up at 6 months after birth	10	10
£25^a^ if ≥ 5 days of Actigraph data recorded	25	NA
Total	£243	£162

NA, not applicable.

^**a**^Shopping vouchers, ^**b**^cash.

#### Treatment group

In addition to behavioral support for smoking cessation, those in the PA group will receive a PA intervention, combining PA consultations and supervised exercise. By extracting the elements of the PA consultation from written manuals and materials provided by the PA program (see Additional file 2; see Additional file 3), the contents of the PA consultation have been classified in accordance with the taxonomy by Michie and colleagues of behavioral change techniques used to help people change their PA behaviors [37]. The researchers delivering the PA intervention will be trained during a 2-day course. There are 14 sessions of supervised exercise, twice a week during the first 6 weeks of the intervention, and then once a week for 2 weeks. During the first 6 weeks, when the smoking-cessation support is provided, one of the exercise sessions will be combined with the smoking-cessation support. Following a familiarization session at the first visit, participants will be advised to aim for 30 minutes of continuous treadmill walking during each session. Following the guidelines [38], moderate-intensity exercise will be prescribed according to age and current activity levels, and monitored using a polar heart-rate monitor. Intensity of exercise will also be guided by the rating of perceived exertion (RPE) [39] (‘fairly light’ to ‘somewhat hard’) and by the ‘talk test’, which indicates that the intensity of activity is too high if the woman cannot hold a conversation.

Twice a week for the first week and then once a week (alternating with smoking cessation support), prior to using the treadmill, the women will receive around 20 minutes of PA consultation (total of nine sessions), aimed at increasing PA undertaken outside the supervised sessions (Table 2). The intervention incorporates 19 of 40 BCTs for increasing PA as defined by Michie *et al*. [37]. The researcher and participant will work through a booklet incorporating the behavior-change techniques (see Additional file 1). The participants will be encouraged to view exercise as a self-control strategy for reducing cigarette cravings and withdrawal [40], and to maintain any increases in PA after their pregnancy. Following recommendations for pregnancy [24,25], the women will be advised to be active for continuous periods of at least 10 minutes at a time, progressing towards accumulating 30 minutes of activity on at least 5 days of the week. The emphasis will be on brisk walking, which is popular among pregnant smokers [41]. As a further option, a home-based antenatal exercise DVD and booklet will be provided. In addition, participants will be given a pedometer (Digi-Walker SW-200; Great Performance Ltd, London, UK) for monitoring their daily steps. Pedometers have been shown to increase activity levels in women [42], and are acceptable during pregnancy [43] and among pregnant smokers [28]. Participants will be asked to log their daily steps, with the therapist calculating a 10% increment every 2 weeks, gradually progressing towards 10,000 steps a day [44].

### Baseline data collection

At baseline, demographic characteristics will be recorded, including age, marital status, number of children, highest educational qualification, ethnicity, occupation, weeks of gestation, and history of premature births. Smoking history will also be recorded, including cigarettes smoked per day (now and before pregnancy), urge to smoke [45,46], tobacco withdrawal symptoms [45,46], and Fagerström Test for Nicotine Dependence score [47], as well as the partner's smoking status. Depression will be assessed with the 10-item Edinburgh Postnatal Depression Scale (EPDS) [48]. Physical activity levels in the previous week will be assessed for both groups using the 7-day Physical Activity Interview [49]. The woman’s confidence about taking up regular PA [50] and stopping smoking [51] will also be reported.

### Outcome measures

#### Primary outcomes

The primary outcome is self-reported continuous abstinence from smoking from the quit date through to the end of pregnancy, validated by either exhaled CO or salivary cotinine levels. This outcome is dichotomous (that is, abstinent or not abstinent). Our definition of continuous abstinence allows a total of five cigarettes after the quit day [52,53]. Expired CO levels will be assessed weekly up to 4 weeks after the quit day and at end of pregnancy using a CO monitor (Smokerlyzer; Bedfont Scientific Ltd, Maidstone, Kent, UK) with a cut-off of <8 ppm [54]. Saliva cotinine levels will be measured at 4 weeks after the quit day and at the end of pregnancy (cut-off level <10 ng/ml) [55]. The aim is to follow-up the woman within 2 weeks of the birth; however, the end-of-pregnancy assessment will be considered valid if recorded between week 36 of pregnancy and 10 weeks after the birth. If a woman’s report of abstinence is disconfirmed by either her CO or cotinine level, she will be considered as having resumed smoking. All the data will be entered on to an online database held on a secure internet server at the CTU at Nottingham University.

#### Secondary outcomes

Continuous smoking abstinence will also be assessed at 4 weeks after the quit day and 6 months after the birth. In addition, we will assess continuous smoking abstinence with a stricter criteria whereby no cigarettes are allowed after the quit day, at 4 weeks after the quit date, and at the end of pregnancy. Again, self-reports of smoking abstinence will be validated by expired CO and cotinine measurements. Self-reports of smoking status at 6 months after the birth will be reported via telephone and will not be biochemically validated. As an incentive, women who complete the follow-up sessions at the end of pregnancy and at 6 months after the birth will be given a £10 shopping voucher for each of these follow-ups. Table 3 summarizes the various financial incentives offered to the participants.

Many women report that, rather than stopping smoking, they reduce their smoking during pregnancy [56,57], and there is some evidence to suggest that a reduction in smoking of 50% or more is associated with increased infant birth weight [58]. Therefore, levels of smoking reduction will be assessed for those women who relapse. Other secondary outcome measures are: changes in urge to smoke, tobacco withdrawal symptoms, and confidence in stopping smoking and in maintaining regular PA. We will also assess changes in depression between baseline, end of pregnancy and 6 months after the birth, as well as changes in maternal weight between baseline, 4 weeks after the quit date, and end of pregnancy.

Further self-reports of PA levels (by 7-day recall) will be collected at weeks 1, 4, and 6 after the quit date and at both follow-ups (that is, end of pregnancy and 6 months after birth). To objectively validate self-reported PA levels, 10% of participants in both the treatment group and the control group will wear an accelerometer (GT1M; Actigraph, Pensacola, FL, USA) for seven continuous days during the fourth week after the quit date. Those who record at least 5 days of Actigraph data will be compensated with a payment of £25 shopping vouchers. The Actigraph has been shown to be practicable and valid during pregnancy [59-61]. Duration of treadmill exercise will be recorded, along with attendance rates at behavioral support and exercise sessions.

The following perinatal measures will be extracted from patient’s hospital records: (i) antenatal complications, including any admissions and the reasons for the admissions, (ii) gestation at onset/induction of labor (and indication for induction where appropriate), (iii) duration of labor and mode of delivery, (iv) Apgar scores of infants, and where available acid–base status of infants, and rates of transfer to the neonatal intensive-care unit, and (v) birth weight and placental weight.

### Cost-effectiveness

From a health-service perspective, we will examine the cost-effectiveness of smoking -cessation support plus exercise relative to smoking-cessation support alone. We will document resources consumed that are related to each intervention. Relevant resources include: cost of personnel, materials, space, equipment, and administrative overheads. Data collection methods include: (i) accounting for staff time using time and effort reports, (ii) accounting for computer time, mailing, and program costs using an accounting system that has been created to facilitate real-time aggregation of these costs, (iii) and using interviews with staff to determine the amount of time they devote to tasks related to the program. The benefits of smoking cessation in pregnancy will be modeled and not measured. Long-term cessation leads to long-term benefits to the mother, which must be discounted appropriately. In ‘normal quitters’ who sustain abstinence for about 6 months, meta-analyses show that half will relapse, while half will become lifetime abstainers and enjoy these benefits [62,63]. Based on this, we might expect that most women who stop smoking in pregnancy, usually for at least 6 months, would maintain lifetime abstinence. However, a much higher proportion of women who stop smoking return to it after pregnancy than would be expected in these models, diminishing the benefits to the woman herself from smoking cessation. However, the benefits of smoking cessation in pregnancy also extend to the fetus, which will be less likely to be born pre-term [18]. This leads to reduced NHS costs and a lower likelihood of complications, such as cerebral palsy and similar health problems. Avoidance of these latter health problems leads to gains in quality-adjusted life years for the baby that must also be included in the utility measure of the decision analytic model. Thus we will use an existing method that has been used for cost-effectiveness modeling for the National Institute for Clinical Excellence (NICE), updating utility estimates to include the benefits to the fetus. The evidence for this model in measuring the benefits to the fetus and child will be derived from a systematic review of the effects of smoking cessation in pregnancy on the consequences for the fetus and child that is currently being conducted by a team at the University of Nottingham, led by Professor Tim Coleman (co-investigator). The analysis will allow us to calculate incremental cost-effectiveness ratios for smoking cessation support plus exercise compared with smoking cessation support alone in terms of cost of treatments per smoker who quits. We will also be able to examine ratios for subgroups; for example, according to nicotine dependence or age at baseline.

### Statistical analysis

We will conduct a descriptive comparison of the baseline characteristics of the two treatment groups. Analysis will be on an intention-to-treat (ITT) basis. Participants who, for any reason, have missing outcome data on the primary outcome or any secondary smoking outcomes, will be assumed to have resumed smoking [55]. Our primary outcome measure, continuous abstinence from smoking from quit date to end of pregnancy, will be compared between treatment groups using logistic regression, adjusted for recruitment center only, with statistical significance determined by the likelihood-ratio test and with the estimate of effect given as the odds ratio and 95% confidence interval. Our primary analysis will not adjust for any further variables because effect estimates can be sensitive to decisions concerning what variables should be adjusted for and how these are specified. However, we will conduct secondary analyses adjusting for the following variables that are predicted to be related to the outcome: education, nicotine dependence, age, and depression. We will analyze other binary smoking outcomes in a similar way.

We will compare secondary outcomes, including urge to smoke, withdrawal symptoms, self-confidence, and PA, in the first week of abstinence, and the same variables plus maternal gestation weight and depression, over subsequent time points, using mixed effects modeling to allow for repeated measures, with adjustment for center. To deal with non-normally distributed variables we will use transformations to normality, residual bootstrapping, or dichotomizing. Differences between groups in perinatal outcomes, including birth weight, gestation, mode of delivery, and complications, will be analyzed by linear or logistic regression, with adjustment for center. For fetal outcomes, the primary analysis will be of singleton births and we will carry out a sensitivity analysis including multiple births, allowing for the clustering of outcomes. If we observe differences between the two groups in use of NRT, or use of behavioral support outside of the intervention sessions, then we will conduct a sensitivity analysis to examine the effect of controlling for any differences between groups in these variables.

We will determine the quantity and distributions of missing data. We will carry out a complete case analysis, and we will compare this with an analysis using multiple imputation to deal with missing values, which assumes data are missing at random, describing any differences in terms of the biases in the data. The exception is smoking outcomes, where those with missing data will be assumed to have resumed smoking [55].

If the intervention is effective, we will use mediation analysis to examine whether there is evidence that the change in PA levels is the likely causal factor in determining smoking abstinence. We will examine the association between treatment group and change in PA levels, and the association between level of PA and abstinence. Finally, we will include level of PA in a logistic regression model of the association between treatment group and abstinence, with mediation assessed using MacKinnon’s causal steps criteria [64].

### Changes to methods since trial enrollment began

Since trial enrollment began on 1 April 2009, the following important changes to the methods have been made.

1)For the follow-up at end of pregnancy, the valid period for assessment was originally defined as 38 weeks gestation to 2 weeks after the birth. Because there were a number of women who could not be contacted to be followed up during this time frame, the valid period was extended to 36 weeks gestation to 4 weeks after the birth (approved by the research ethics committee on 18 May 2010). However, because there were still a few women being followed up later than 4 weeks after the birth, the valid period was further revised to 36 weeks gestation to 10 weeks after the birth (approved 31 Jan 2012). The aim remains to attempt to follow-up as many women as possible within 2 weeks of the birth.

2)To give the women an incentive to complete the follow-ups at end of pregnancy and 6 months after the birth, all women who complete these follow-ups will be given a £10 shopping voucher for each of the follow-up sessions attended (approved 18 May 2010).

3)Originally, to be eligible women had to report smoking at least 10 cigarettes a day before their pregnancy. We found that a good number of women reported smoking five to nine cigarettes a day at this time. Therefore, we extended the eligibility criteria to include women smoking at least five cigarettes a day before pregnancy (approved 15 September 2010). These women are still likely to be dependent on smoking, as there is evidence that women who say they were smoking five to nine cigarettes before pregnancy are back to smoking 14 cigarettes a day at 18 months post-partum [65].

4)Initially, women had to be between 12 and 24 weeks' gestation to be eligible for the trial. However, since the trial began, most of the hospital trusts began offering earlier antenatal booking appointments (before 12 weeks gestation), and because we wished to recruit the women as early as possible in pregnancy, we revised this to 10 to 24 weeks gestation (approved 31 Jan 2012).

## Discussion

Because NHS Stop Smoking Services provide individual behavioral support to pregnant women as a standard treatment, we will be testing an intervention that could be readily introduced into current NHS practice and might be expected to cost less per patient than conventional treatments, such as NRT. This study has been designed to address many of the limitations highlighted in previous research examining PA as an aid for smoking cessation [15], by including both supervised exercise and PA consultations, being adequately powered, using a remote randomization system to protect concealment of allocation, and adopting an ITT approach to the analysis. If found to be effective, physical activity could be an important alternative or adjunctive treatment for smoking cessation during pregnancy, particularly for women who prefer non-pharmacological interventions. Currently, however, little is known about the mechanisms that might mediate any therapeutic effects of PA on smoking cessation [15]. There are a number of hypothesized biological and psychosocial mechanisms, but it is likely that an effective PA intervention would rely on multiple mechanisms. Future research might identify which particular mechanisms, and any interactions between them, are most important, and determine the optimum type, intensity, and duration of PA required to produce a therapeutic effect.

## Current study status

The LEAP trial began recruiting patients in April 2009, and recruitment will close in November 2012. Data collection for the primary outcome is due to be completed in July 2013. As of October 2nd 2012, 768 women have been recruited.

## Abbreviations

BCTs: Behavior Change Techniques; CTU: Clinical trials unit; GP: General practitioner; ITT: Intention-to-treat; LEAP: London Exercise And Pregnant smokers; NICE: National Institute for Clinical Excellence; NRT: Nicotine replacement therapy; NHS: National Health Service; PCT: primary care trust; RCT: Randomized controlled trial; RPE: Rating of perceived exertion.

## Competing interests

The authors declare that they have no competing interests.

## Authors’ contributions

All authors were responsible for the initial protocol, securing funding for the trial, and refinement of the protocol. MU is both the chief investigator and the trial manager. Data analysis will principally be conducted by MU with the assistance of SL. MU wrote the initial draft of the manuscript. All authors contributed to and approved the final manuscript.

## Supplementary Material

Additional file 1 Therapist manual for delivering smoking cessation intervention. Click here for file

Additional file 2 Therapist manual for delivering physical activity intervention. Click here for file

Additional file 3 Participant’s handbook for physical activity intervention. Click here for file
