# Supplementary material for: Physical activity as an aid to smoking cessation during pregnancy (LEAP) trial: study protocol for a randomized controlled trial
Source: Trials. 2012 Oct 4;13:186. doi: 10.1186/1745-6215-13-186 (PMC3519528; doi:10.1186/1745-6215-13-186)
Supplement: Additional file 1 — Therapist manual for delivering smoking cessation intervention. [file 1745-6215-13-186-S1.pdf]

Smoking cessation support manual for LEAP trial

This manual includes guidance all 43 behaviour change techniques (BCTs), except BM4 "Provide rewards contingent on successfully stopping smoking", defined in the following taxonomy: Michie S, Hyder N, Walia A, West R (2011) Development of a taxonomy of behaviour change techniques used in individual behavioural support for smoking cessation. Addictive Behaviour, 36, 315-9. The 43 BCTs used are:
Specific focus on the target behavior (*B*) and maximizing motivation (*M*): BM1. Provide information on consequences of smoking and smoking cessation; BM2. Boost motivation and self-efficacy; BM3. Provide feedback on current behavior; BM5. Provide normative information about others' behavior and experiences; BM6. Prompt commitment from the client there and then; BM7. Provide rewards contingent on effort or progress; BM8. Strengthen ex-smoker identity; BM9. Identify reasons for wanting and not wanting to stop smoking; BM10. Explain the importance of abrupt cessation; BM11. Measure CO. **Maximizing self-regulatory capacity and skill (*BS*)**: BS1. Facilitate barrier identification and problem solving; BS2. Facilitate relapse prevention and coping; BS3. Facilitate action planning/develop treatment plan; BS4. Facilitate goal setting; BS5. Prompt review of goals; BS6. Prompt self-recording, BS7. Advise on changing routine; BS8. Advise on environmental restructuring; BS9. Set graded tasks; BS10. Advise on conserving mental resources; BS11. Advise on avoiding social cues for smoking. **Promoting adjuvant activities (*A*)**: A1. Advise on stop-smoking medication; A2. Advise on/facilitate use of social support; A3. Adopt appropriate local procedures to enable clients to obtain free medication; A4. Ask about experiences of stop-smoking medication that the smoker is using; A5. Give options for additional and later support. **General aspects of interaction focusing on delivery of the intervention (*RD*)**: RD1. Tailor interactions appropriately; RD2. Emphasize choice; **General aspects of interaction focusing on information gathering** (RI): RI1 Assess current and past smoking behaviour; RI2. Assess current readiness and ability to quit; RI3. Assess past history of quit attempts; RI4. Assess withdrawal symptoms; General aspects of interaction focusing on general communication (RC): RC1. Build general rapport; RC2. Elicit and answer questions; RC3. Explain the purpose of CO monitoring; RC4. Explain expectations regarding treatment program; RC5. Offer/direct toward appropriate written materials; RC6. Provide information on withdrawal symptoms; RC7. Use reflective listening; RC8. Elicit client views; RC9. Summarize information/confirm client decisions; RC10. Provide reassurance.

The specific BCTs used are indicated in brackets in the manual below. The version of the manual used by therapists did not include these BCT labels.

Each smoking cessation consultation should take about 20 minutes.

Session 1: Preparation for quitting

Briefly describe the study and intervention and check volunteer's understanding:

- **Explain randomisation** e.g. 'A computer will decide whether you will get the standard treatment or the exercise programme. Do not be too concerned about which you are getting. Whatever you receive, you are in with a good chance of succeeding in stopping smoking. In both conditions we shall be providing help and support to help you to stay free of smoking.'

- **Remind them what the interventions will involve (RC4)** e.g. 'If you are in the exercise group you will need to attend 14 appointments over 8 weeks. This will involve waking on a treadmill for up to 30 mins and advice and support with stopping smoking and taking regular exercise. If you receive the standard treatment you will be asked to attend 6 weekly appointments providing advice and support with stopping smoking.

- **Explain the timing of the target quit date** (TQD): We will ask you to stop smoking one week from today. **(BS4)**

- The volunteer should not be randomised using the online database until you are satisfied that she understands what the study will entail (especially the need to attend all appointments) and understands the process of randomisation.

IF RANDOMIZED:

- **Explain the purpose of carbon monoxide (CO) monitoring (RC3)**
- **Measure CO (BM11) and use the reading to motivate quitting.**
  e.g. 'This shows how much you inhale the smoke and all the dangerous chemicals in it. A non-smoker would normally have a reading of less than eight. You as a smoker have a reading of ___. As soon as you stop smoking, this will start to go down to the non-smoking level. This will happen not just in your own body, but in the body of your baby as well. There is an immediate health benefit in stopping smoking. We shall repeat this measure once a week.'

- **Briefly discuss reasons for wanting to quit, level of motivation for quitting and confidence for quitting** (refer to questions in CRF) **(BM9, R12)**

- Assess and discuss current and past smoking behavior (see questions in CRF) (**RI1**)

- **Discuss past attempts at quitting (R13)** and reasons for relapse. Determine longest periods of abstinence and, if these are reasonably lengthy, use these as a reason to be optimistic to about being able to quit.

- **Discuss preparing for the quit day (BM6, BS3)**; e.g. "I suggest you make a list of the things you need to do to prepare for your quit day. For example: Tell people you are quitting, particularly those who will give you support **(A2)**, decide when you will have your last cigarette, tell people that they will not be allowed to smoke in the home, remove all ashtrays and from your home **(BS8)**, become aware of times when you are most likely to lapse (e.g. after meals, when alone in the house, on phone) and make a note of these **(BS6)**, think about how you will deal with these times and be aware that one puff can easily turn into a full relapse **(BS1)**." Also ask them to think about whether there are any events coming up that might make it difficult not to smoke (e.g., a stressful event or party, or meeting someone they used to smoke with) **(BS1)**. Mention that there will be more time to discuss these strategies on their quit day.

- **Discuss whether partner smokes** and whether partner or friends or colleagues might want to quit with them. Invite partner to join them at their treatment sessions. **(A2)**

- **If they have children**: discuss what they will do with children during the sessions. Also, suggest they explain to the children that they are quitting and that they ask the children to encourage them. **(A2)**

- Explain clearly that the woman can have the last cigarette before the session on their quit day (e.g. outside the clinic before coming in, on the morning or the night before their quit day. No smoking after that) **(BS4)**. If they can, ask them to decide now when they would like to have their last cigarette and say that it is their choice **(RD2).**

- **Give a leaflet about smoking and smoking cessation during pregnancy (BM1, RC5)**

- If they ask **whether they should start cutting down** in preparation for quitting, explain that it is better not to start missing cigarettes before the real quit attempt begins. This is likely to be a better way to quit than having long breaks between the last few cigarettes, enjoying them greatly, and saying 'goodbye ciggies' with tears in their eyes and having strong cravings before they have even quit.

- **If volunteer says they are content with cutting down to 2 or 3**, consider mentioning the following to encourage them to quit abruptly and completely **(BM10)**
- . It is often anxiety and discomfort that motivates behaviour change rather than logic:

**Encouraging Women to Quit Completely**
Not much benefit to be had by cutting down as you inhale more deeply and more nicotine reaches the baby. You have the worst of both worlds as you are experiencing the discomfort of cutting down and doing the same harm to yourself and the baby.
You will be adding more stress making those few cigarettes more precious and there is also the stress of withdrawal between those few cigarettes. Better to quit completely.
It is best to quit completely now because the pressure that comes with having a new baby will make it more likely that you will relapse.

- If they ask about **self-rolled and low tar cigarettes** explain that these are just as harmful. **(BM1)**

Session 2: Quit Day

Objectives

- Remind patient that today is their quit day
  Look for reasons why woman is a good prospect (BM3) (e.g. managed to quit for a period of time in the past, she is highly motivated, lessons learned from previous attempts, success/change in other areas of their life, is receiving best available support etc.). Express your optimism. Make it clear that they need to be prepared for it being very difficult (particularly if quitting was difficult in the past, CO is high and/or they smoke their first cigarette soon after waking), but that many women in their position quit successfully

- **Stress the importance of a good start**. e.g. 'You have shown determination by getting as far as this. Now is the crunch time. Even if the first few days prove to be difficult, do not go back to smoking. As a long-term smoker, you need to expect at least some difficult moments. The good news is that there is strong evidence that making it through the first week without a single puff massively increases your chances of success. Please remember this when you are tempted to smoke.

- **Explain the withdrawal symptoms** that they may experience. 'Besides cravings, many people feel irritable, depressed, restless, have poor concentration, sleep disturbance and feel hungrier.' Also some people get more colds, mouth ulcers and headaches. **(RC6).** Reassure that these will gradually decrease across the first 3-4 weeks of quitting. The first week often is the hardest and the middle of the first week is often the worst time.

- **Smoking and stress:** Explain that smoke increases rather than reduces stress and that most people feel less stress within a week or two of quitting.

- **If they are ambivalent about quitting** remind them of why they want to quit.

- **Discuss possible obstacles to success and briefly review coping strategies** for week ahead: Ask 'What happened when you tried to stop smoking before? Refer woman to tips in the leaflets. Ask "Are there any particular events this week that might increase your temptation to smoke?" **(BS1)**
  Explain that cravings often only last a few minutes and that they tend to peak and then subside. Recommend that they prepare for these cravings **(RC6)** (e.g. distract yourself, have an activity ready, for exercise group suggest a short walk, have someone you can call when you are close to lapsing, keep healthy snacks in your bag (e.g. apples, carrot sticks) and water (some people say that sipping helps with cravings), have things to keep your hands occupied, avoid/minimize difficult/stressful/tempting situations if you can (e.g. parties, time spent with smokers,), especially in the first few weeks **(BS11)**. If they cannot avoid these situations suggest they be prepared for it to be a challenge. Explain that it can also be useful to change their daily routine (e.g. if they usually smoke first thing in the morning suggest they replace this with something else, such as a shower or breakfast). **(BS7)** Suggest that they also conserve their energy, particularly in the first few weeks, by not taking on major new projects such as making other significant changes in their life **(BS10)**.
- A typical tempting situation which may lead to smoking is when people are bored with not much to do. If this affects you, make plans for distracting yourself. To give you an example, Mrs. Palmer found that for her, making a jigsaw helped. She started a jig-saw puzzle on her quit day and when tempted to smoke went and looked for the next piece of her jigsaw.

- **Suggest cognitive strategies (RC6)** . Some people benefit from 'talking themselves through' difficult situations; e.g. remind yourself that the difficult moment will soon pass, remember your reasons for stopping smoking and the danger of having even one slip, think of all you've achieved so far, think how you will feel if you lapse and have a cigarette, write down all the reasons why you don't want to smoke and keep this with you, keep a picture of the baby's scan with you.
  - 'If tempted to smoke, have a look at the tips in this leaflet. Distract yourself if it gets tough. Take it one day or even one hour at a time. **(BS9)**
  Remember that if you pull through this week without smoking at all, you are far more likely to succeed.

- If they make these preparations this will **build up their resistance** when they are tempted to smoke.

- **Finish by stressing the importance of not having a single puff of a cigarette** as this can quickly lead to having more cigarettes and it will increase your cravings. You may feel that having just one cigarette won't hurt and will make you feel better. Be on your guard! This is how attempts at quitting fail. Do not fool yourself. **(BS2)**

Session three : one week after quit day

Main aims :
- **Assess CO** and give feedback about whether their reading has reduced **(BM11, BM3).**

- **How do I handle discrepancies between CO and self-report?**
  - Best not to challenge self-reports, but check again that client did not smoke
  - Check ambient CO
  - Check your own reading
  - Ask about the possibility of exposure to secondary tobacco smoke.
  - Ask about possibility of exposure to CO at home (e.g. leaky gas boiler)
  - Say that you will calibrate the CO monitor and check their reading again again next week

- **Review** how they got on this week with remaining abstinent and discuss whether the strategies they prepared were helpful. **(BS5)**

- **Boost motivation**, provide orientation on withdrawal, discuss possible reasons for relapse and strategies for dealing with these **(BS2)**.

- **Reinforce abstinence**, deal with lapses.

Check smoking status. If abstinent, praise and reinforce: e.g. **"**If you did not smoke at all, you are now free of nicotine and of many other chemicals in cigarette smoke which are bad news for your baby" (**BM7**).

- **If they lapsed,** explain again the importance of complete cessation, but do not discourage (e.g. 'You still have time to catch up. How do you plan to go about it?'*).* Discuss a plan. Look for a positive angle. Explain to lapsers who complain of withdrawal discomfort that this will only go away if they do not smoke at all – see below. Check for unrealistic expectations ('e.g. Despite the exercise/NRT, I still fancied a cigarette'). **If they have relapsed** back to regular smoking encourage them to learn from why they relapsed and to set a new quit date **(BM6)**. If they have relapsed, discuss whether they would consider using NRT **(RD2)** and provide **advice about NRT (A1)**. Liaise with the PCT to enable them to get free NRT **(A3)**.

- **Discuss withdrawal discomfort** and refer to their responses to the withdrawal questions in the CRF **(R14)**. Reassure where appropriate *(*e.g., 'Over many years, your brain and body have got used to having regular shots of nicotine throughout your waking hours. Once you stop smoking, it can take weeks and months to regain your balance.'*).* Explain the likely duration of concrete symptoms patients are concerned about. Reassure them that most smokers experience strong withdrawal symptoms and that cravings are the most common symptom **(BM5, RC10)**; e.g. "They can be unpleasant, but they will not harm you. You will be over the most difficult time soon". Suggest they continue to be aware of, or make a note of, when they are most tempted to smoke **(BS6)**.

- **Discuss triggers for relapse and coping efforts (BS2)** where appropriate. Encourage them to use: distraction techniques (I go and do something, get away from the situation, etc.), avoiding temptations (I do not have any cigarettes in the house, did not go to the pub this week), and cognitive approaches (I think of my reasons for stopping, tell myself to take one day at a time, I imagine how bad/guilty I will feel if I smoke etc.).

- Suggest they start **seeing themselves as a non-smoker**, rather than as a smoker who is trying to quit or an ex-smoker. If they do this they are more likely to succeed. (e.g. if someone offers them a cigarette suggest they say 'No thanks, I don't smoke', and ask them to imagine saying this) **(BM8)**.

- Allow time at the end of each session for the **participant to ask questions (RC2)**.

- Remind participant that if they attend all six smoking cessation sessions they will be entered into a **prize draw** for three £100 shopping vouchers. **(BM7)**

Sessions 4 (two weeks after quitting) onwards

Main aims:
Reinforce abstinence or deal with lapses.
Give reassurance on withdrawal (at later sessions deal with weight gain).
Discuss obstacles, advise on coping, express support.
Tailor the content for each individual **(RD1)**

- **Check smoking status (BS5).** Congratulate abstainers and praise them. Explore how they got through difficult moments; elicit, reinforce, and develop coping strategies. Note they are now making it (first 2 weeks) or have made it through the most difficult part of quitting.
  If not abstinent, discuss barriers to quitting. Explain the urgency of catching up.

- **If a participant is smoking** daily, debrief on difficulties that led to smoking, and suggest lessons learned and future solutions (**BS2**). Help patient decide whether to continue current quit effort or to set a new quit date (**BM6**). Frame the experience positively, and focus on future efforts. Offer to refer them to the PCT for further support during or following the research intervention **(A5)**.

- **Discuss triggers for relapse and coping strategies (BS2)**

- **If you go through a difficult patch**, remember that it will get easier soon. Your brain and body got used to regular doses of nicotine. It can take a few weeks, or more, to get back your balance and to learn to live without smoking. Most people feel OK within some three or four weeks.

- **Ask about use of NRT**: refer to the questions in the CRF and, for those who are using NRT, ask how they are getting on with it. **(A4)**

- **Emphasise the rationale of recommending complete abstinence** and the danger of relapse.

- By now, you have probably **saved quite a bit of money** you would otherwise spend on cigarettes. This saving will grow quickly. Plan how to spend it on yourself.

FU at six weeks after quitting: remember that you **need to conduct a 7 day physical activity recall via telephone for control group 6 weeks after quit.**

- Explain the danger of relapse and the need to stay on guard. 'Many smokers who make it through pregnancy go back to smoking after the birth or within a year of quitting. I would like to make sure that this will not happen to you'. (**BS2**)

- Warn against 'transgression' cigarettes when stressed, when bored, on holidays, and in company. Discuss the temptation people often have 'to try one cigarette, just to see', explain the dangers, especially in the hours and days after the birth. **(BM8, BS2)**

- Discuss likely relapse situations (e.g. smokers they will come in contact with soon after the birth) and plan coping strategies. (**BS2**)

- Emphasise woman's ability to cope and implement cessation procedures on their own, building on the success to date.

- The woman may be concerned about what will happen after the visits finish. In case they need to contact you, give them your clinic phone number. Offer them the option of being referred on to the PCT stop smoking service for further support. Give them information about the NHS website for pregnant smokers (www.smokefree.nhs.uk/smoking-and-pregnancy/) **(RC5)** and the smokefree helpline: 0800 022 4332. **(A5)**

- If they say they feel tempted in the company of smoking friends/family, suggest they consider asking these individuals not to smoke around them, or just plan how to react when one of them lights up or offers a cigarette. (**BS2**)

- "One of these days you will realise that you have not thought of smoking at all for hours. Notice this. It will soon become days and then weeks, until cigarettes will stop being an issue at all."

- "Every day you make it without smoking, you are learning how to cope without cigarettes and finding your own ways to overcome difficult moments. Notice what helps and use it."

- "The link between smoking and everyday activities such as getting up in the morning, speaking on the phone, or having a meal will by now be weakening – notice such positive developments."

- "If you still have difficult moments, do not fall into the trap of thinking that one cigarette will not hurt. Even a puff could put you in serious danger of undermining all you have achieved so far." (**BS2, BM8**)

- "Try to eat a healthy, balanced diet. This includes plenty of fruit and vegetables, and not too much fried and fatty food. Keep a supply of healthy snacks with you. Watch out for high calorie, high fat snacks, but do not go hungry."

Tips for preventing relapse (BS2)

If you have not smoked at all, or very little, for over four weeks now, you are really getting there. Once the regular support is over, here are some tips to prevent you going back to smoking:

- Think ahead of situations which could be dangerous for you. Boredom? Stress? Getting drunk? Wanting to enjoy yourself? Think now how you are going to cope. When the situation comes, remember how you prepared for it.

- Do not think that after being a non-smoker for a few months, one cigarette will not make any difference. It will be as dangerous as ever.

- Even if things are still difficult on occasions, sooner or later, you will lose interest in smoking, or even start to dislike the very idea of it. You are very close now to join millions of others who have stopped smoking for good.

Some women who quit smoking successfully when pregnant start to smoke again after they give birth. Stay on your guard.

- Passive smoking is harmful for your baby.
- Quitting smoking is very difficult. You have now done the hard part. It would be a shame to spoil it all.
- Sooner or later you will start thinking of yourself as a non-smoker and the idea of smoking will start to look rather strange and less appealing.

Notes on the support process (this also applies to physical activity counselling)

Key things to remember:

- **Summarise** information the women needs to remember **(RC9)**.
- **Reflective listening**: reflecting back to the women what they are saying, particularly the emotional content (e.g. I can appreciate that it makes you angry and frustrated to see your partner continuing to smoke around the house when you have quit). **(RC7)**
- **Acknowledge their fantasies** about smoking, but explain why they are unrealistic (e.g. Yes, I can see that you would love to have the occasional cigarette, but smokers who do this very quickly start smoking on a daily basis back to their original levels of smoking). In general, try and explore their views on smoking cessation (**RC8**).
- **Descriptive praise**: describe what they are specifically doing well at and praising them for this (e.g. you did well to throw all your cigarettes away because I know you partly wanted to keep some for an emergency), rather than just using general praise (e.g. you are doing very well). **(BM4)**
- **Preparing for success**: Focus on helping the women to plan so that things go right (e.g. preparing for triggers to smoking), rather than having to 'react' when things go wrong **(BS1)**.
- **Maintaining a positive tone** e.g. avoid criticism, use pleasant tone of voice and body language, focus on solutions rather than problems, keep your sense of humour! **(RC1)**

For those familiar with psychological counselling, it is important to note that brief smoking cessation support is much more directive and goal-oriented than general counselling approaches.
