# Supplementary material for: Physical activity as an aid to smoking cessation during pregnancy (LEAP) trial: study protocol for a randomized controlled trial
Source: Trials. 2012 Oct 4;13:186. doi: 10.1186/1745-6215-13-186 (PMC3519528; doi:10.1186/1745-6215-13-186)
Supplement: Additional file 2 — Therapist manual for delivering physical activity intervention. [file 1745-6215-13-186-S2.pdf]

Physical Activity Intervention Manual for LEAP Study

(refer also to participant's handbook: Additional File 3)

This manual includes guidance on 19 of 40 behaviour change techniques (BCTs) defined in the following taxonomy: Michie S, Ashford S, Sniehotta FF, Dombrowski SU, Bishop A, French DP (2011) A refined taxonomy of behaviour change techniques to help people change their physical activity and healthy eating behaviours: the CALO-RE taxonomy. Psychological Health, 2, 1479-98.
The 19 BCTs covered in this manual are: 1. Provide information on consequences of behaviour in general; 2. Provide information on consequences of behaviour to the individual; 7. Action planning; 8. Barrier identification/problem solving; 9. Set graded tasks; 10. Prompt review of behavioural goals; 12. Prompt rewards contingent on effort or progress towards behaviour; 13. Provide rewards contingent on successful behaviour; 16. Prompt self-monitoring of behaviour; 20. Provide information on where and when to perform the behaviour; 21. Provide instruction on how to perform the behaviour, 22. Model/Demonstrate the behaviour; 23. Teach to use prompts/cues; 26. Prompt practice; 27. Use of follow-up prompts; 29. Plan social support/social change; 34. Prompt use of imagery; 35. Relapse prevention/coping planning; 38. Time management.

The specific BCTs used are indicated in brackets in the manual below. The version of the manual used by therapists did not include these BCT labels.

Each smoking cessation consultation should take about 20 minutes.

Session 1 (one week before quit date)

● **Review her current PA levels** (refer to seven day recall of physical activity).

● **Explain how to use treadmill (BCT 21:** Instruction on how to perform behaviour**)**
- Explain warm-up (3mins walking, hold stretches for 10 secs for front thighs, calves, hamstrings and reach overhead for upper body) and warm down (slow down for final minute of walking, repeat stretches)
- Recommend rating of perceived exertion of 12-14 and show RPE chart. Explain about 'Talk Test', and that exercise should be intense enough for her to be breathing heavier than normal
- Demonstrate use of treadmill (**BCT 22**: Demonstrate behaviour)
- Ask women to walk on treadmill for 15-30 mins
- Agree how long she will walk (it is fine if she exceeds this goal, as long as it is no more than 30 mins)

● **Discuss benefits of exercise (BCT 1:** Provide information on consequences of behaviour in general**)**
- Mention that regular exercise has been shown to reduce cravings in a similar way to nicotine replacement.
- Mention that exercise is also good for a healthy pregnancy. Say that there will be more time to discuss these benefits at the next session.

● **Agree PA goals for this week (BCT 7:** Action planning**)** for exercise she will do outside the treadmill Sessions:

- Recommend she starts with at least one session of 15 mins PA.
- Recommend that she gradually progresses towards 30 mins of PA on 3 to 5 days a week (plus treadmill sessions).
- Agree a SMART goal, e.g. ‘I will walk for 20 mins around the park at lunchtime, on 5 days this week’.

● **Explain how to use pedometer**
- Ask her to wear pedometer for the rest of today and to take it off last thing at night and then to open it and record the number of steps in the diary.
- Each morning, ask her to put pedometer on as soon as she gets up and to wear it all day. Again, taking it off last thing at night and recording the number of steps (**BCT 26**: Prompt practice). Say that she can keep the pedometer.

● **Ask her to complete PA and steps diary** for everyday this week (**BCT 16**: Self-monitoring of behaviour). Write her PA goal for this week on the top of the diary.

Session 2 (few days before quit day)

● **Review goals/plans**
- Check whether she has managed to do any PA in her own time since the last session
- Briefly review woman's goals for taking PA in her own time for week ahead (**BCT 10:** review of behavioural)
- Check she has been able to use the pedometer OK and is recording her daily steps. If she is averaging less than 10,000 steps a day recommend a 10% increase in her current steps over the next two weeks (**BCT 7:** action planning, **BCT 9:** set graded tasks).

● **Go though physical activity booklet with woman**
- Ask her to write down what she sees as the main benefits and disadvantages (if any) of becoming more active during her pregnancy and remind her of other benefits (**BCT 2**: Consequences of behaviour to the individual).
- Testing times: ask her to write down any barriers that might prevent her from achieving her PA goal and think of ways of overcoming these barriers (**BCT 8:** Barrier identification problem solving).
- Go though the tips for exercising in the booklet and praise her for any specific adjustments she has made to her lifestyle to encourage exercise (**BCT 12**: Rewards contingent on effort or behaviour). Suggest that she rewards herself when she achieves her exercise goals (e.g. a special meal at the end of the week) (**BCT 13**: Reward contingent on successful behaviour).
- Demonstrate the home exercises in the booklet and ask her to try each of the level 1 exercises/stretches with you. **(BCT 21:** Instruction on how to perform behaviour, **BCT 22**: Demonstrate behaviour). Encourage her to practice the exercises at least once before the next meeting (**BCT 26**: prompt practice). When she is confident with the level 1 exercise, go through the Level 2 exercises/stretches at a future session.
- Provide information on local opportunities for exercise (e.g. walking schemes, antenatal exercise classes) (**BCT 20:** information on where and when to exercise).
- Encourage her to exercise at regular times (e.g. a walk after lunch) so that it becomes a habit (**BCT 23:** Teach to use prompts/cues). If she has raised lack of time as a barrier to exercise, suggest she manages her time to fit in exercise by timetabling exercise slots into her week (**BCT 38**: time management).
- Suggest she tries to find people who will exercise/walk with her (**BCT 29**: Plan social support).

● **Ask woman to walk on treadmill for 15-30 mins**
- If she walked for less than 30 mins at the last session recommend that she walks for 5 mins longer this time and at each further session, until she is walking continuously for 30 mins.

- Agree how long she will walk for.
- After exercise: Remind woman to complete PA and steps diary every day this week.

Session 3 (quit day)

● Briefly review physical activity the women has done in her own time and adjust goals for physical activity in general and for pedometer (**BCT 10:** review of behavioural goals)

● Discuss any barriers that might prevent her from achieving her PA goal and think of ways of overcoming these barriers (**BCT 8:** Barrier identification problem solving).

- Praise her for any specific adjustments she has made to her lifestyle to encourage exercise (**BCT 12**: Rewards contingent on effort or behaviour). Discuss how she is rewarding herself when she achieves her exercise goals (**BCT 13**: Reward contingent on successful behaviour).

● Set heart-rate zone and ask woman to wear HR monitor while on treadmill:
HEART RATE TARGETS/(training zone):

Less active (ie reporting less than 150 mins PA in the last week), overweight (BMI=25 – 29.9) or obese (BMI=30+) women:
Start with light intensity heart-rate range:
Aged 16-29: 102 -124 bpm
30 years plus: 101-120 bpm

Gradually progress to moderate intensity HR range:
Aged 16 -29: 125 -144 bpm
30 years plus: 121-144 bpm

Active women (ie reporting at least 150 mins/week):
Moderate intensity HR range:
Aged 16-29 145-160 bpm
Aged 30 plus 140-156 bpm

● Ask woman to walk on treadmill for 15-30 mins

● Ask woman to check that she keeps her heart rate in the training zone, and ask her to maintain her walking at a level where she is still able to hold a conversation

● Give exercise diary and ask her to fill it in each day this week.

Consultation Session 4 (one week after quit day) onwards

● Review physical activity the women has done in her own time:
- Discuss barriers that may prevent her maintaining and increasing her physical activity and how she might deal with these (**BCT 35**: relapse prevention/planning)
- Agree plans for PA for the coming week:
- Refer back to physical activity booklet if necessary

● Praise her for any specific adjustments she has made to her lifestyle to encourage exercise (**BCT 12**: Rewards contingent on effort or behaviour). Discuss how she is rewarding herself when she achieves her exercise goals (**BCT 13**: Reward contingent on successful behaviour).

● Review whether she has found people who will exercise/walk with her.

● Review exercises in booklet.

● Ask woman to walk on treadmill for 15-30 mins

● Ask her to imagine herself becoming fitter and healthier and to imagine herself walking briskly with energy and with lungs free of tobacco (**BCT 34**: use of imagery)

● Remind her that for the last two weeks of the exercise programme you will see her once a week rather than twice a week (**BCT 27**: use of follow-up prompts).

Notes on the support process

Key things to remember:

- **Summarise** information the women needs to remember
- **Reflective listening**: reflecting back to the women what they are saying, particularly the emotional content (e.g. I can appreciate that it is very difficult to find the time to exercise).
- **Descriptive praise**: describe what they are specifically doing well at and praising them for this (e.g. you did well to buy some new trainers; this shows that you are serious about taking more physical activity), rather than just using general praise (e.g. you are doing very well).
- **Preparing for success**: Focus on helping the women to plan so that things go right (e.g. preparing for when it rains), rather than having to 'react'.
- **Maintaining a positive tone** e.g. avoid criticism, use pleasant tone of voice and body language, focus on solutions rather than problems, keep your sense of humour!
