# Supplementary material for: Physical activity as an aid to smoking cessation during pregnancy (LEAP) trial: study protocol for a randomized controlled trial
Source: Trials. 2012 Oct 4;13:186. doi: 10.1186/1745-6215-13-186 (PMC3519528; doi:10.1186/1745-6215-13-186)
Supplement: Additional file 3 — Participant’s handbook for physical activity intervention. [file 1745-6215-13-186-S3.pdf]

LEAP

Name:…………………………………………

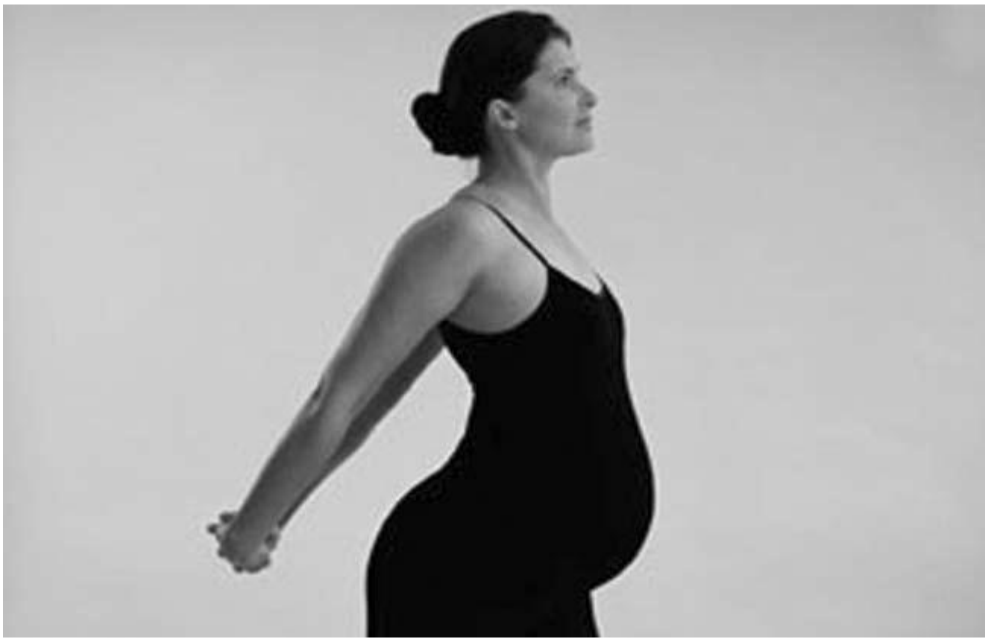

Guide to physical activity during your pregnancy

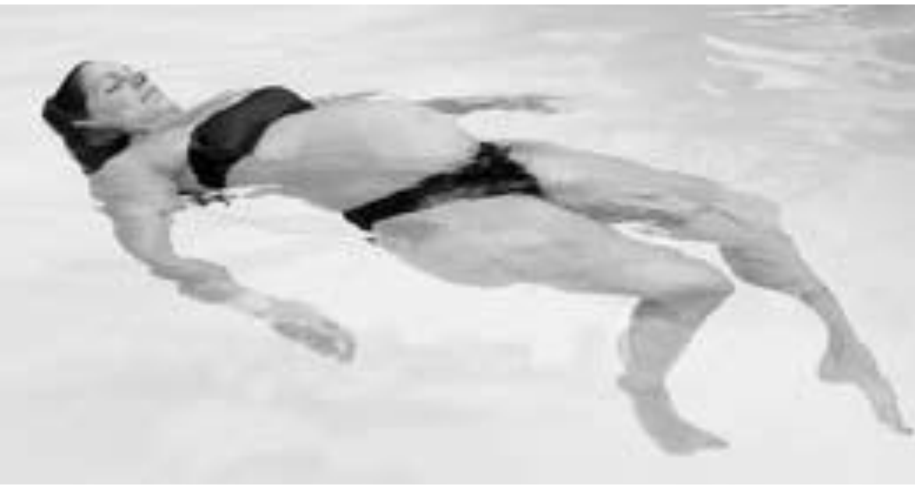

BENEFITS

Regular physical activity during your pregnancy has many benefits for you and your baby; such as:

- Less varicose veins, leg cramps and swelling of the legs
- Better posture, balance and muscle tone
- Reduced constipation and back ache
- Easier and shorter labour, in many women
- Better mood, sleep and energy
- Less tobacco withdrawal symptoms (such as feeling irritable) and cravings, and an increased chance of becoming a non-smoker

Write down what you feel are the main benefits and disadvantages for you of becoming more active during your pregnancy:

BENEFITS	DISADVANTAGES
______________________________	______________________________
______________________________	______________________________
______________________________	______________________________
______________________________	______________________________
______________________________	______________________________
______________________________	______________________________
______________________________	______________________________

- Can you think of any way of overcoming the disadvantages?
- If you think of any more benefits add them to the list.

- <u>Set a realistic goal</u> each week for how much activity you can manage each day

MY GOAL:

……………….. minutes per day

- <u>Aim</u> to gradually build up to 30 minutes of activity each day.

TESTING TIMES

- Write down anything that might stop you from achieving this goal
- Try and think of what you can do when this happens

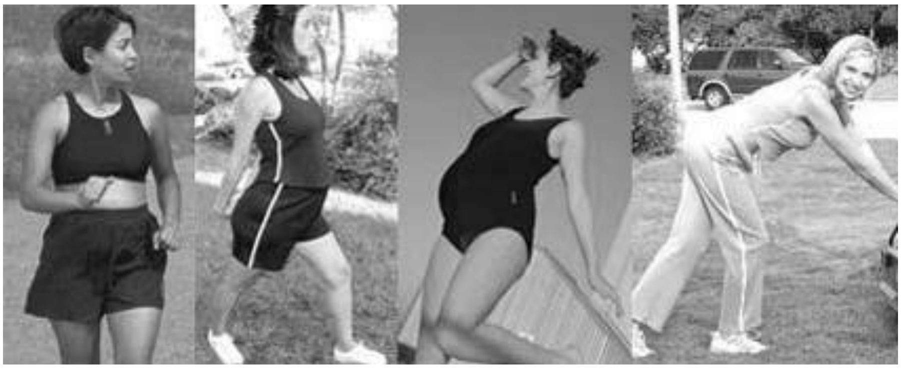

Why I might miss a days exercise	What I can do when this happens.

SOME TIPS

1. Don't smoke, walk!
When you feel a strong craving for a cigarette try going for a brisk walk, even if it is only for 5 minutes. Soon you will start feeling like a non-smoker.

2. Make it Fun
- Choose activities which you enjoy, and which fit easily into your day. Try walking part of the way to or from work or the shops. How about swimming or following your home exercise routine?
- Play music while you're exercising
- Involve friends or family

3. Do it every day
- Gradually increase the number of days on which you are active
- Then increase the time you are active for each day
- Gradually build up to 30 minutes of physical activity each day

4. Make it a habit
- Exercise at the same times each day, so that it becomes a habit
- Try starting the day with a few gentle exercises or going for a walk

5. Take it easy
- Choose activities that make you breathe slightly harder than normal, but are not hard enough to stop you having a conversation
- Do just a little more (10-20 minutes extra) each week
- Give your body time to adjust to being pregnant and to being without cigarettes

6. Reward yourself
- Each extra day that you are a non-smoker and have reached your exercise goal give yourself a treat
- Give yourself an extra treat at the end of each week; something like a special meal.

7. Plan ahead
Plan ahead for interruptions such as bad weather - an ideal opportunity to try your home exercise routine!

8. Keep a diary
Keep a daily record of how many minutes exercise you do and how many steps you do. It will help you to see your progress

LOOK AFTER YOURSELF!

1. When to avoid exercise
- If you feel unwell, extremely tired or have just eaten a meal
- In very hot or very humid conditions

2. When to stop exercising
Stop exercising immediately if you feel any dizziness, nausea, severe pain or tiredness, extreme breathlessness or cold sweats

3. Breathe Freely
- Keep breathing freely whilst you are exercising
- To ease breathing: stop and lift your arms up and out

4. Drink plenty of water
Before, during and after exercise

5. Have a healthy snack soon after exercising, such as a banana.

6. Avoid exercising on your back.
- During the middle and later stages of your pregnancy exercising on your back can cause discomfort.
- It may also reduce your blood pressure for a short time

7. Pace yourself
Start slowly and gradually work up to a pace where you are breathing slightly heavier than normal, but not gasping for breath

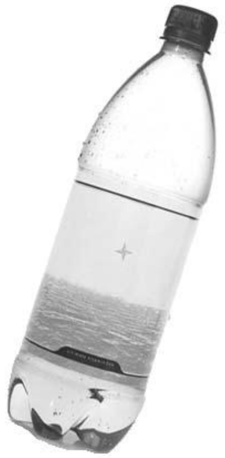

HOME EXERCISE PROGRAMME

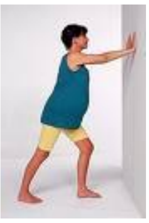

1. **Build up gradually**
   - For each exercise aim for a number of repetitions which you can handle comfortably
   - Aim to gradually build up the number of repetitions you do, until you can do 20 or more
   - If you want to exercise for longer you can gradually build up to two or more complete circuits of all the exercises

2. **Taking breaks**
   - At first you may made need a short break between exercises
   - Gradually reduce the length of the breaks, so that eventually you do the exercises continuously

3. **To avoid aggravation or injury:**
   - Start slowly. Give your body time to warm-up
   - Avoid locking your knees when you are standing
   - Keep good posture, with a straight back.
   - Finish by doing some stretches.

4. **Moving up a level**
   Start with the exercises at level 1. When you are comfortable with level 1 move on to level 2.

If you exercise at home as opposed to in a class you can choose when, how and at what pace you exercise!
Alternatively, you may prefer to exercise in a class in which case you should inform the teacher that you are pregnant

LEVEL 1 EXERCISES

- <u>Try starting with 5 repetitions</u> of each exercise. Over several weeks, gradually build to 20 repetitions
- <u>Do at least 10 minutes of exercise</u> by going through all the exercises several times

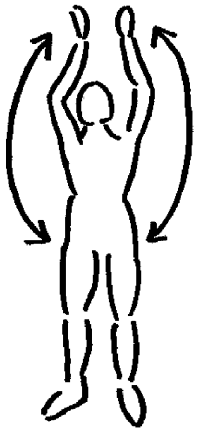

1. Side arm raise

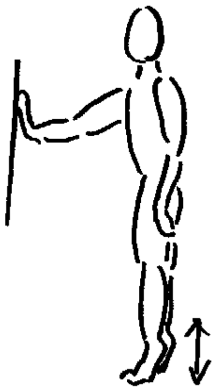

2. Raise on toes

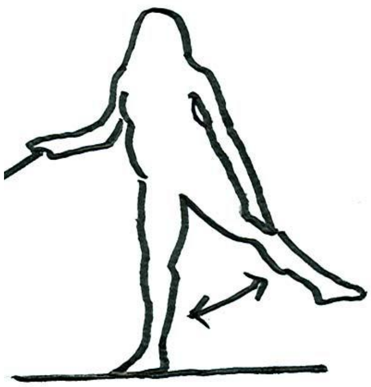

3. Side leg raise

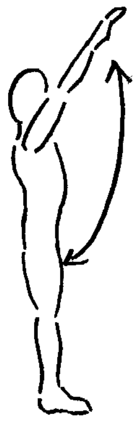

4. Front arm raise

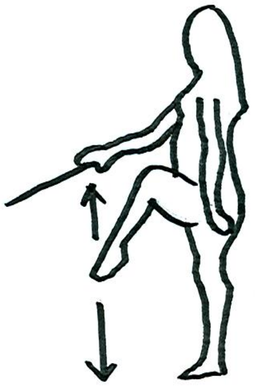

5. Knee raise

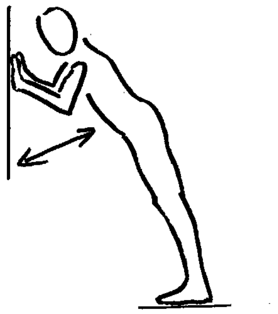

6. Wall-push up

LEVEL 1 STRETCHES

- Stretch regularly to help you feel loose and relaxed. Stretching also will help you with your posture and balance during pregnancy
- Stretch when you are warm, after you have done your other exercises
- Hold the stretch so that you feel a pleasant stretch, do not force the stretch. Avoid any bouncing movements.

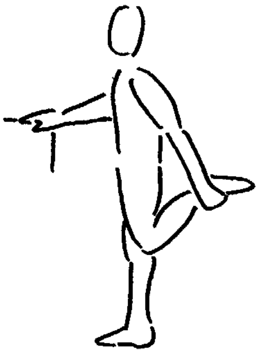

1. Pull heel to buttock.
(for front of thigh)

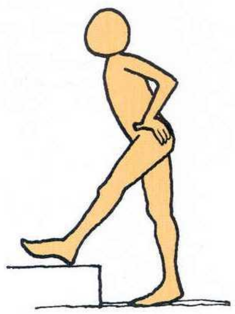

2. Lean over straight leg
(for back of thigh)

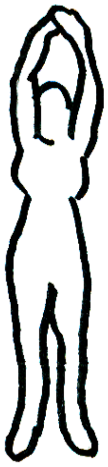

3. Reach arms overhead.
(for arms and sides of body)

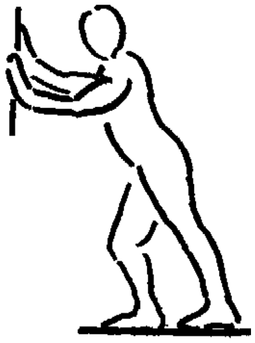

4. Lean on wall, straighten back leg.
(for calves)

Hold each stretch for 10 seconds

LEVEL 2 EXERCISES

Here are some slightly harder exercises for you to gradually build in

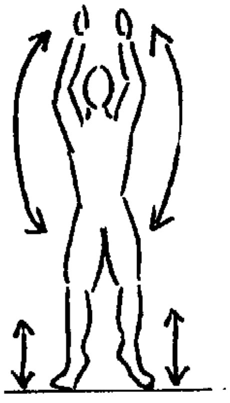

1. Side arm raise + toe raise

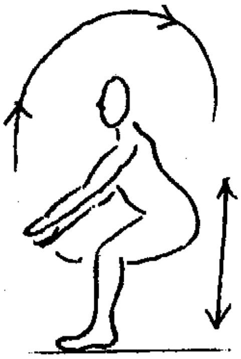

2. Backwards arm circle + leg bend

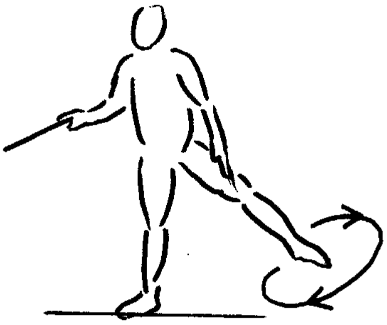

3. Leg circle

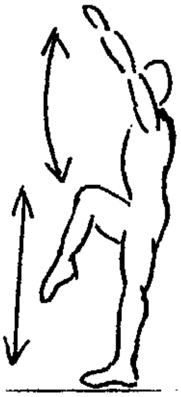

4. Front arm raise + knee raise

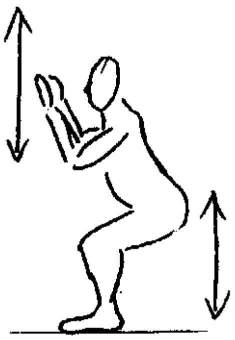

5. Overhead-arm reach + leg bend

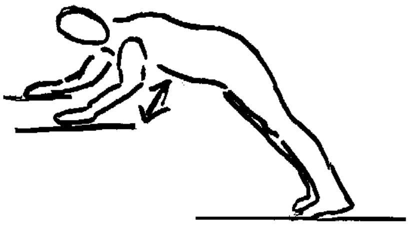

6. Push-up on chair

LEVEL 2 STRETCHES

Here are some extra stretches

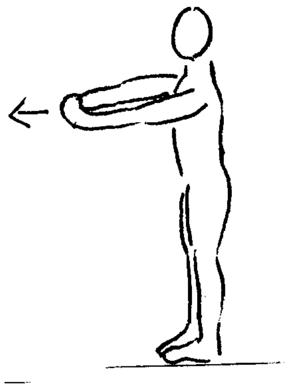

1. Pull arms forward
(for upper back & shoulders)

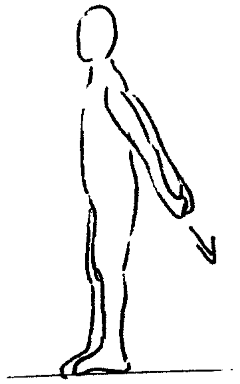

2. Pull arms back
(chest & shoulders)

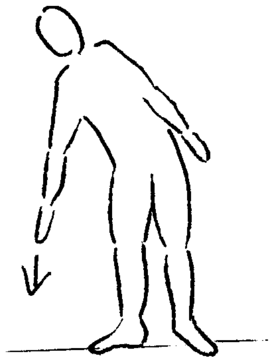

3. Bend to side
(waist)

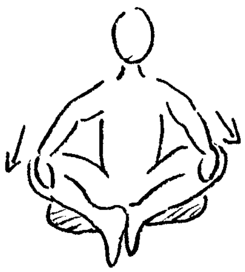

4. Press down on inner thighs
(inner thighs)

Hold each stretch for 10 seconds
